# Identification of a copper-responsive small molecule inhibitor of uropathogenic *Escherichia coli*

**DOI:** 10.1128/jb.00112-24

**Published:** 2024-06-10

**Authors:** Braden S. Hanson, Amanuel Hailemariam, Yongjian Yang, Faras Mohamed, George L. Donati, Dwight Baker, James Sacchettini, James J. Cai, Sargurunathan Subashchandrabose

**Affiliations:** 1 Department of Veterinary Pathobiology, College of Veterinary Medicine and Biomedical Sciences, Texas A&M University, College Station, Texas, USA; 2 Department of Biochemistry and Biophysics, College of Agriculture and Life Sciences, Texas A&M University, College Station, Texas, USA; 3 Department of Veterinary Integrative Biosciences, College of Veterinary Medicine and Biomedical Sciences, Texas A&M University, College Station, Texas, USA; 4 Department of Chemistry, Wake Forest University, Winston-Salem, North Carolina, USA; Queen Mary University of London, London, United Kingdom

**Keywords:** *E. coli*, UPEC, copper, isothiazolone, antimicrobial

## Abstract

Urinary tract infections (UTIs) are a major global health problem and are caused predominantly by uropathogenic *Escherichia coli* (UPEC). UTIs are a leading cause of prescription antimicrobial use. Incessant increase in antimicrobial resistance in UPEC and other uropathogens poses a serious threat to the current treatment practices. Copper is an effector of nutritional immunity that impedes the growth of pathogens during infection. We hypothesized that copper would augment the toxicity of select small molecules against bacterial pathogens. We conducted a small molecule screening campaign with a library of 51,098 molecules to detect hits that inhibit a UPEC Δ*tolC* mutant in a copper-dependent manner. A molecule, denoted as *E. coli* inhibitor or ECIN, was identified as a copper-responsive inhibitor of wild-type UPEC strains. Our gene expression and metal content analysis results demonstrate that ECIN works in concert with copper to exacerbate Cu toxicity in UPEC. ECIN has a broad spectrum of activity against pathogens of medical and veterinary significance including *Acinetobacter baumannii, Pseudomonas aeruginosa*, and methicillin-resistant *Staphylococcus aureus*. Subinhibitory levels of ECIN eliminate UPEC biofilm formation. Transcriptome analysis of UPEC treated with ECIN reveals induction of multiple stress response systems. Furthermore, we demonstrate that L-cysteine rescues the growth of UPEC exposed to ECIN. In summary, we report the identification and characterization of a novel copper-responsive small molecule inhibitor of UPEC.

**IMPORTANCE:**

Urinary tract infection (UTI) is a ubiquitous infectious condition affecting millions of people annually. Uropathogenic *Escherichia coli* (UPEC) is the predominant etiological agent of UTI. However, UTIs are becoming increasingly difficult to resolve with antimicrobials due to increased antimicrobial resistance in UPEC and other uropathogens. Here, we report the identification and characterization of a novel copper-responsive small molecule inhibitor of UPEC. In addition to *E. coli*, this small molecule also inhibits pathogens of medical and veterinary significance including *Acinetobacter baumannii, Pseudomonas aeruginosa,* and methicillin-resistant *Staphylococcus aureus*.

## INTRODUCTION

Urinary tract infections (UTIs) are a significant global health problem with approximately 150 million cases annually (1, 2). Uropathogenic *Escherichia coli* (UPEC) is the most prevalent causative agent of UTI (2, 3). Other Gram-negative and Gram-positive pathogens can also colonize and infect the urinary tract, specifically individuals who are at high risk for developing a UTI (2–4). Colonization of the kidney can lead to pyelonephritis, which is a major risk factor for developing bacteremia or septicemia (2, 3, 5). Antimicrobials play a central role in clinical management of UTI.

Antimicrobials such as β-lactams, sulfonamides, or fluoroquinolones are used to treat UTI (3, 6). However, continued and widespread use of these drugs has led to the emergence of antimicrobial resistance against these first-choice agents (3, 6–12). Although antimicrobial resistance is increasing, there has been limited progress in new antimicrobial drug discovery since 1980s (5, 13–16). Current projections indicate that antimicrobial-resistant infections could emerge as one of the most significant causes of mortality by 2050 (12). Therefore, there is an urgent need to identify and develop next-generation antimicrobial agents to combat infectious diseases. Nutritional immunity has emerged as a key component of the innate immune response against bacterial pathogens. Briefly, effectors of nutritional immunity either sequester essential nutrients (Fe, Mn, and Zn) from the pathogens or promote toxicity (Cu and Zn) to the invading pathogens (17–20). Copper is of special interest because of its historical use for antimicrobial properties, and is mobilized to sites of infection to impede bacterial growth and modulate several immunological processes (21–28). However, antimicrobial agents in the drug development pipeline do not appear to interact with copper (16).

Here, we report the findings of a small molecule screen for antimicrobials to detect copper-dependent inhibition. We hypothesized that copper would augment the toxicity of select small molecules against bacterial pathogens. We used uropathogenic *E. coli* as the primary target in our studies because of the known involvement of copper in mitigating bacterial colonization during UTI (28–30). We describe the detection of an isothiazolone, which we denote as *E. coli* inhibitor (ECIN) in this manuscript and its additive activity with copper to inhibit UPEC. Additionally, ECIN has a broad spectrum of activity against many pathogens of public health significance.

## RESULTS

### High-throughput screening led to the identification of ECIN

**FIG 1:**
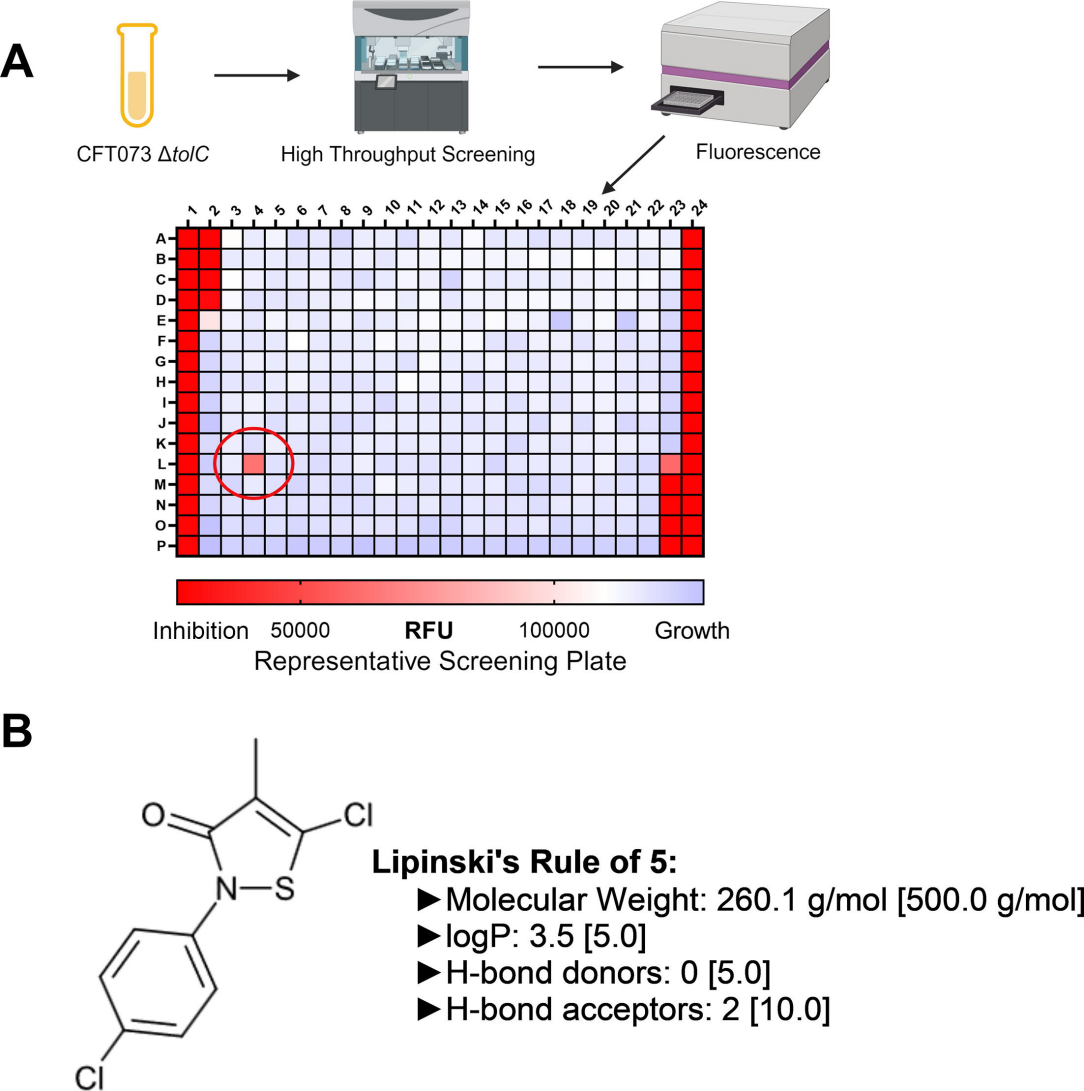
Design of high throughput screening and identification of ECIN. (**A**) Frozen stocks of UPEC strain CFT073 *ΔtolC* were thawed and diluted in LB media. An Analytik Cybio media handler was used to add media, drugs, cells, and resazurin into the wells. Plates were incubated for 24 h and then fluorescence was measured. A representative image of a screening plate is pictured above. Columns 1, 2, 23, and 24 were used for controls. Each screening plate had ~320 different small molecules. Red indicates complete bacterial inhibition while blue indicates complete bacterial growth. A representative hit is indicated by the circle. (**B**) Structure of 5-chloro-2-(4-chlorophenyl)−4-methyl-2,3-dihydro-1,2-thiazol-3-one the hit identified in our screen was named as ECIN and further characterized in this report. ECIN meets the Lipinski’s rule of 5 for drug-like properties, and the cutoff values for these criteria are indicated in the brackets.

A 51,098 small molecule library was screened against the uropathogenic *E. coli* strain CFT073 Δ*tolC* grown in LB supplemented with 25 µM copper to mimic the level of copper found in the blood (31). We used a Δ*tolC* mutant to increase the likelihood of identifying inhibitors because TolC is a major efflux pump involved in resistance to xenobiotics (32). Metabolic activity in the presence of small molecules was inferred from resazurin reduction. The mean relative fluorescence unit (RFU) for the control wells for each screening plate was calculated, and then each of the test well was assigned an individual z-score in relation to the plate mean. The primary screen revealed that 488 molecules (0.95% of the library) inhibited UPEC growth (Fig. 1A). During a secondary screen, primary screen hits (488 compounds) were tested for a significant copper-dependent change in 50% inhibitory concentration (IC_50_). A total of 192 molecules (0.37% of the library) exhibited a copper-responsive decrease in IC_50_ of 1.0 µM or greater, determined as described in Materials and Methods. Additionally, only 12 molecules (0.02% of the library) inhibited the Δ*tolC* mutant growth in a dose-dependent manner. We identified a compound, 5-chloro-2-(4-chlorophenyl)−4-methyl-2,3-dihydro-1,2-thiazol-3-one (named as *E. coli* inhibitor and shortened to ECIN), that inhibited growth of wild type UPEC CFT073 (Fig. 1B). ECIN met/exceeded the criteria for small molecules with drug-like properties described by Lipinski et al. (33), and was characterized further.

### ECIN exhibits additive interaction with sub-lethal concentrations of copper against UPEC

**FIG 2:**
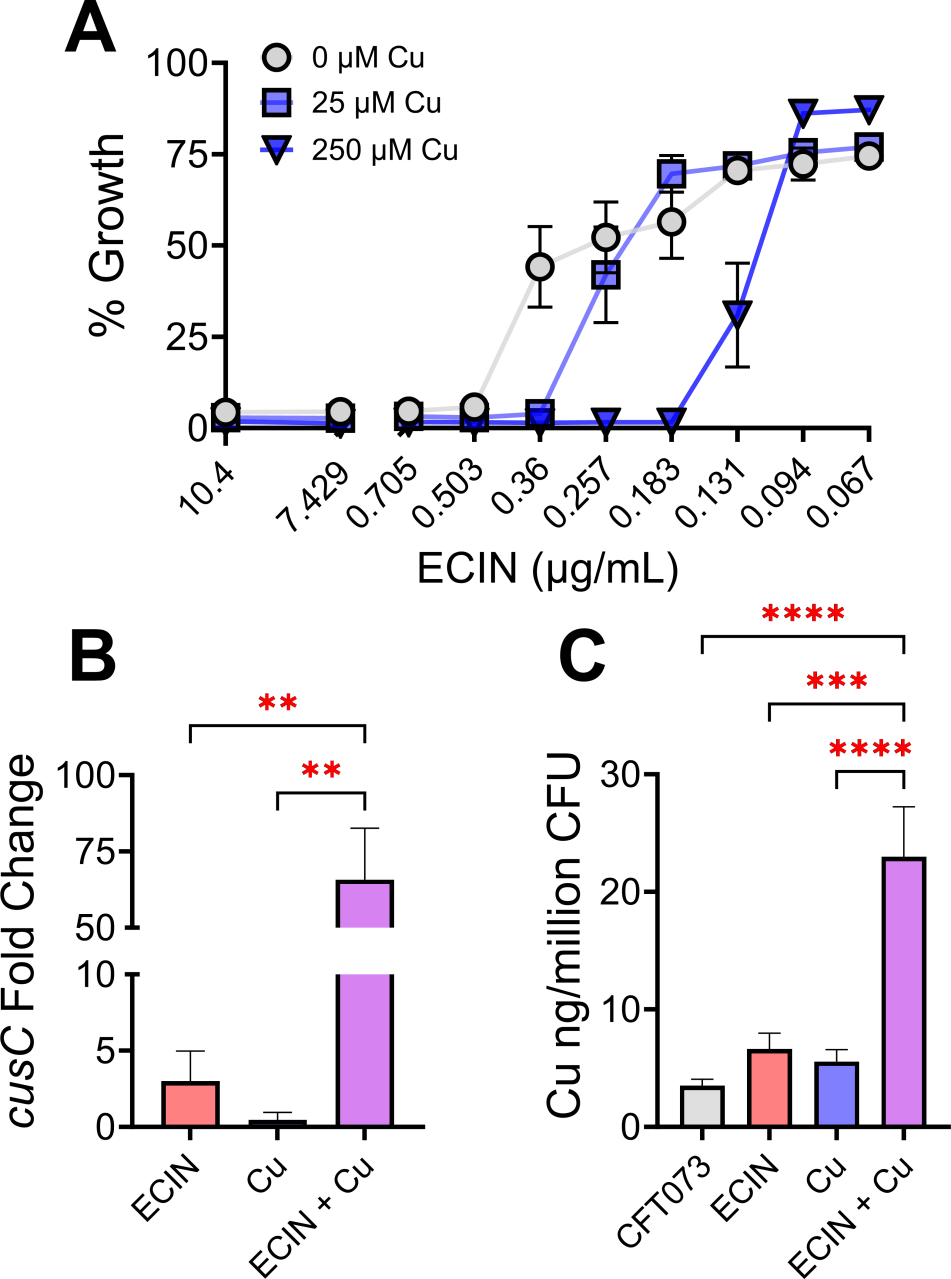
Interaction between ECIN and copper. (**A**) IC of ECIN against UPEC decreases in the presence of copper. Metabolic activity of UPEC treated with varying concentrations of ECIN with or without copper was determined by measuring the fluorescence of resorufin and used to calculate % growth. Mean with SEM are represented (*n* = 9). (**B**) ECIN magnifies the copper efflux response in UPEC. The expression of major copper efflux gene, *cusC*, was assessed in UPEC when treated with ECIN (1.3 µg/mL; 5 µM), copper (5 µM), and the two in combination (*n* = 9). Fold-change relative to untreated wild-type strain is presented ANOVA with Bonferroni, **P < 0.01. (**C**) ICP-MS revealed that ECIN increased the amount of cell-associated copper in UPEC (*n* = 9). ANOVA with Bonferroni, ***P < 0.001 and ****P < 0.0001.

To confirm the interaction of ECIN with copper, checkerboard assays were performed to determine the fractional inhibitory concentration indices (FICI, Fig. S2). The FICI scores were calculated as previously defined (34, 35). The FICI scores were as follows: for ECIN plus CuSO_4_, 0.584 ± 0.02, and for ECIN plus CuCl_2_, 0.509 ± 0.05, indicating an additive interaction. To further evaluate the relationship between ECIN and copper, kill curve assays were performed with various concentrations of copper (Fig. 2A). The IC_50_ of ECIN in the absence of copper was 336 ng/mL but decreased to 260 and 128 ng/mL in the presence of 25 and 250 µM copper, respectively, confirming the results from our checkerboard assays (Fig. 2A).

### ECIN induces copper stress by increasing the cell-associated copper content in UPEC

Quantitative PCR was used to measure the abundance of the *cusC* transcript, encoding a major copper efflux pump in UPEC, because the expression of *cusC* gene is upregulated in UPEC during copper stress (28, 30). At a sub-inhibitory concentration, copper increased the expression of *cusC* by 0.5-fold compared to an untreated control (Fig. 2B). ECIN alone was notably stronger than copper and induced a threefold increase in *cusC* transcript levels compared to an untreated control (Fig. 2B). Most interestingly, when ECIN plus copper was present, there was a statistically significant, 65-fold increase in the *cusC* gene transcript abundance compared to an untreated control (Fig. 2B). Viable counts were determined under the same culture conditions used for the qPCR assay to ensure that data were not skewed by changes in bacterial viability (Fig. S3). To distinguish copper-activated transcriptional response from changes in cellular copper content in UPEC, inductively coupled plasma mass spectrometry (ICP-MS) was used to measure the cell-associated copper concentrations and other key transition metals (Fe, Zn, and Mn). ICP-MS results revealed that there was significantly more cell-associated copper when ECIN plus copper was added as opposed to individual treatments or untreated control (Fig. 2C). When UPEC was treated with ECIN in combination with copper, iron, zinc, and manganese levels were also elevated. However, iron and manganese were the only metals that revealed a statistically significant increase in the presence of ECIN and copper compared to controls (Fig. S4). Results from zone of inhibition assays also support the exacerbation of Cu stress by ECIN in UPEC and non-UPEC *E. coli* strains (Fig. S5A and B). Collectively, our data indicate that ECIN is a potent inhibitor of UPEC that works additively with copper to maximize copper toxicity in UPEC.

### ECIN has a broad spectrum of activity and is bactericidal against UPEC

**FIG 3:**
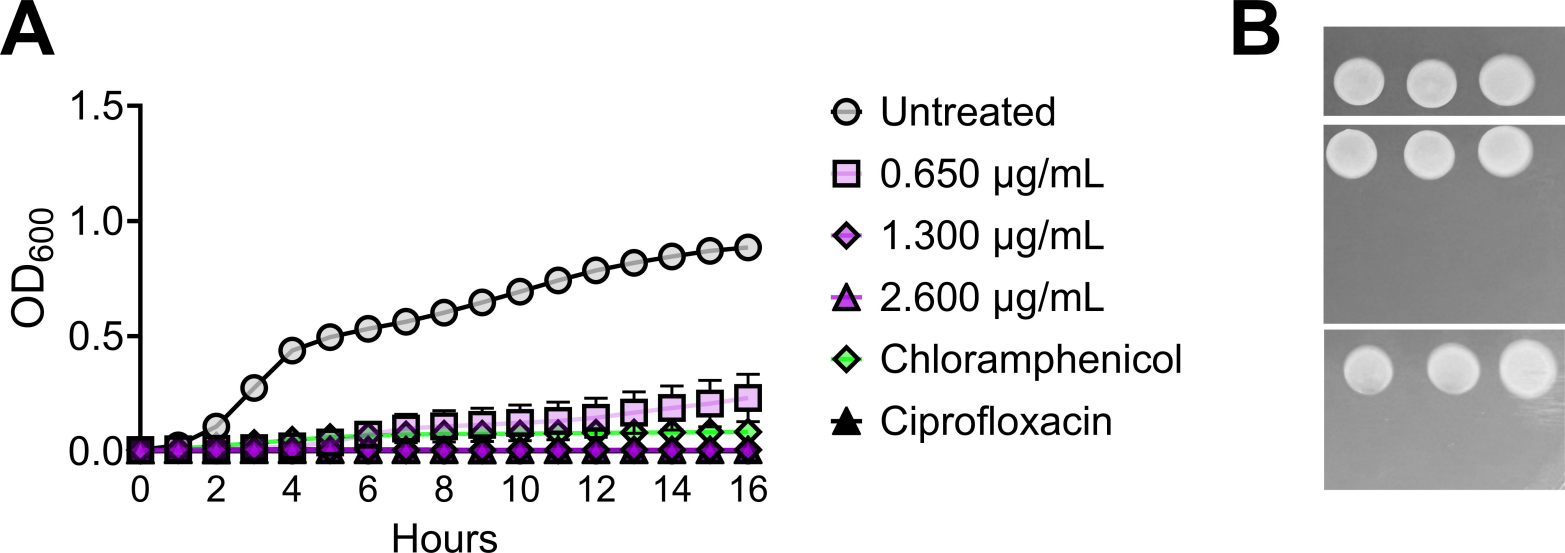
ECIN is bactericidal against UPEC. (**A**) Optical density of UPEC CFT073 cultures containing ECIN or controls was measured over time. Chloramphenicol (MIC = 8 µg/mL) and ciprofloxacin (MIC = 32 ng/mL) were used as bacteriostatic and bactericidal controls, respectively (*n* = 9). Error bars represent SEM. (**B**) The cultures from the growth curve were spot plated on LB agar to determine the presence of viable bacteria.

Broth microdilution assays performed against various gram-negative pathogens and methicillin-resistant *Staphylococcus aureus* strains revealed that ECIN has a broad spectrum of activity and has comparable minimum inhibitory concentrations (MICs) against a diverse array of pathogens (Table 1). ECIN was also effective by inhibiting UPEC in different enriched media, sterile human donor urine, and minimal media used (Table S3). Notably, ECIN was more effective, and copper-dependent killing was observed only in the minimal medium (Table S3). We also performed zone of inhibition assays to test whether Cu increased the effectiveness of ECIN against non-UPEC uropathogens. Our findings clearly indicate that Cu increases the activity of ECIN against a broad range of uropathogens (Fig. S5C and D). Kinetic growth assays of UPEC strain CFT073 were performed with ECIN at 0.5×, 1×, and 2× MIC with controls (bacteriostatic-chloramphenicol and bactericidal-ciprofloxacin). When UPEC strain CFT073 was treated at MIC or higher, the growth curve was identical to that of the bactericidal antibiotic, ciprofloxacin (Fig. 3A). Growth in the presence of subinhibitory concentration of ECIN was comparable to that of the bacteriostatic agent, chloramphenicol (Fig. 3A). Spot plates revealed that viable UPEC was recovered only from cultures with subinhibitory concentrations of ECIN and the bacteriostatic control (Fig. 3B). Another prototypical clinical UPEC isolate UTI89, and an antimicrobial-resistant UPEC isolate HM69 (resistant to ampicillin and trimethoprim sulfamethoxazole) also showed identical results (Fig. S6).

**TABLE 1:** MICs of ECIN against various pathogens

Pathogen	Strain	ECIN	
		μg/mL	μM
Enterobacterales			
*E. coli*	CFT073	1.300	5.000
	UTI89	1.300	5.000
	HM16	0.650	2.500
	HM60	1.300	5.000
	HM69	1.300	5.000
*Klebsiella pneumoniae*	KP4	1.300	5.000
*Proteus mirabilis*	HI4320	1.300	5.000
*Serratia marcescens*	WF191	1.300	5.000
Pseudomonadales			
*Acinetobacter baumannii*	ATCC 17987	0.325	1.250
*Pseudomonas aeruginosa*	ATCC 27312	1.300	5.000
	ATCC 27583	1.300	5.000
	ATCC 49198	0.650	2.500
Bacillales			
*S. aureus*	SF8300, USA 300	0.650	2.500
	SA116, USA 300	0.650	2.500

### ECIN prevents biofilm formation but does not eradicate existing biofilms

**FIG 4:**
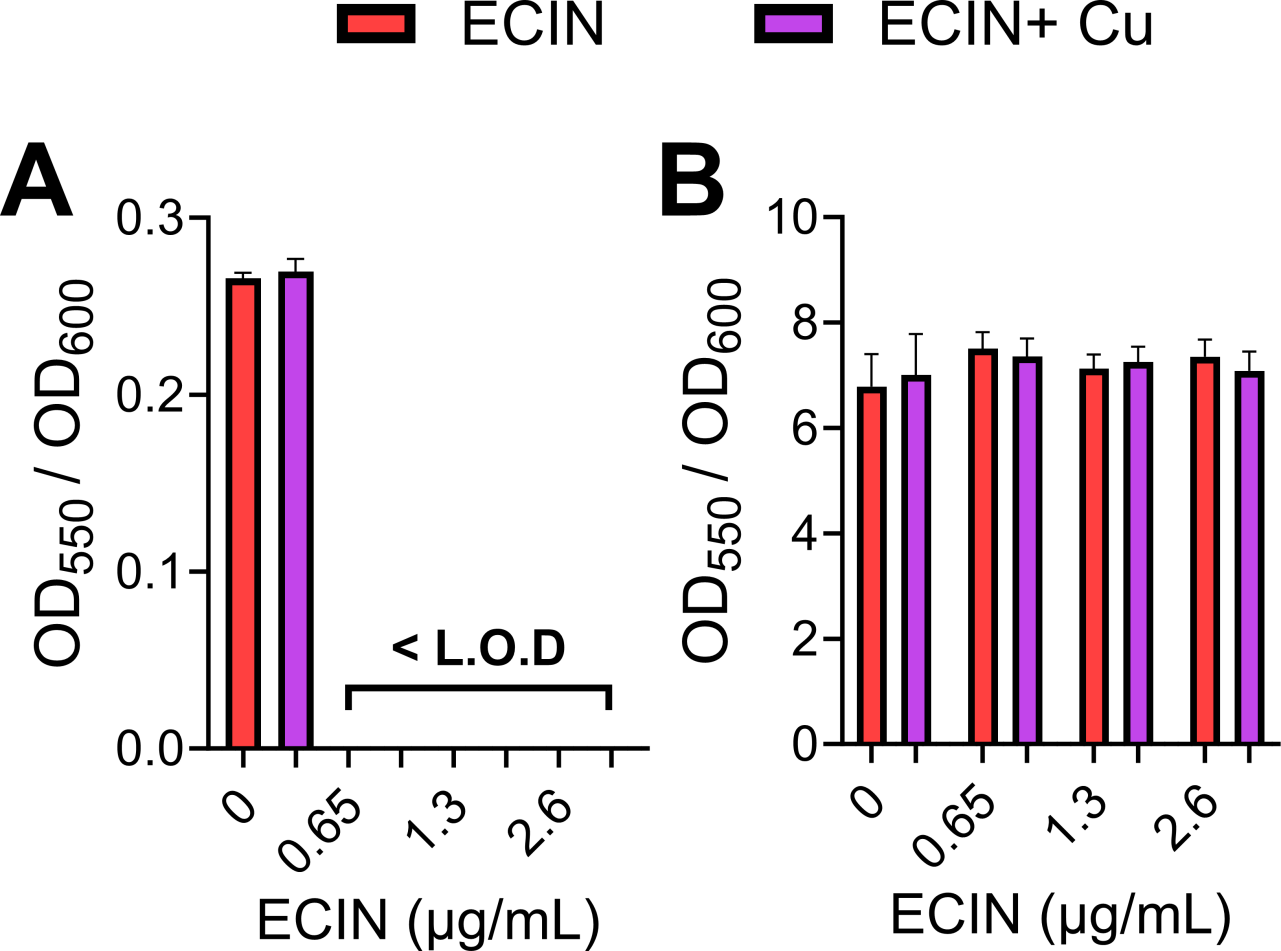
ECIN prevents the formation of biofilms but is unable to reduce pre-existing biofilms. (**A**) Biofilm prevention assays. The ratio of biomass (OD_550_) formed to growth (OD_600_) when UPEC was inoculated with ECIN. (**B**) Biofilm eradication assays. The biomass to growth ratio of pre-formed biofilms after treatment with ECIN for 24 h. No statistical significance was detected (ANOVA with Bonferroni). Error bars represent SEM (*n* = 9).

The UPEC strain UTI89, the prototypical UPEC strain used for studying biofilms, was utilized to assess whether ECIN could prevent or eradicate biofilms. Biofilm biomass was stained with crystal violet (36). Biofilm prevention assays (Fig. 4A) revealed that bacteria treated with ECIN at 0.5×, 1×, and 2× MIC were unable to form a quantifiable biomass. Interestingly, while growth was apparent at 0.5× MIC (Fig. S6A and B), biofilm biomass was below the limit of detection (Fig. 4A). For biofilm eradication assays, biofilms were established before the addition of ECIN (Fig. 4B). There was no significant change in the biomass normalized to growth between the ECIN-treatment and controls (Fig. 4B). The addition of copper to the media had no effect on the ability of ECIN to prevent or eradicate biofilms (Fig. 4A and B).

### ECIN upregulates several bacterial stress response systems in UPEC

**FIG 5:**
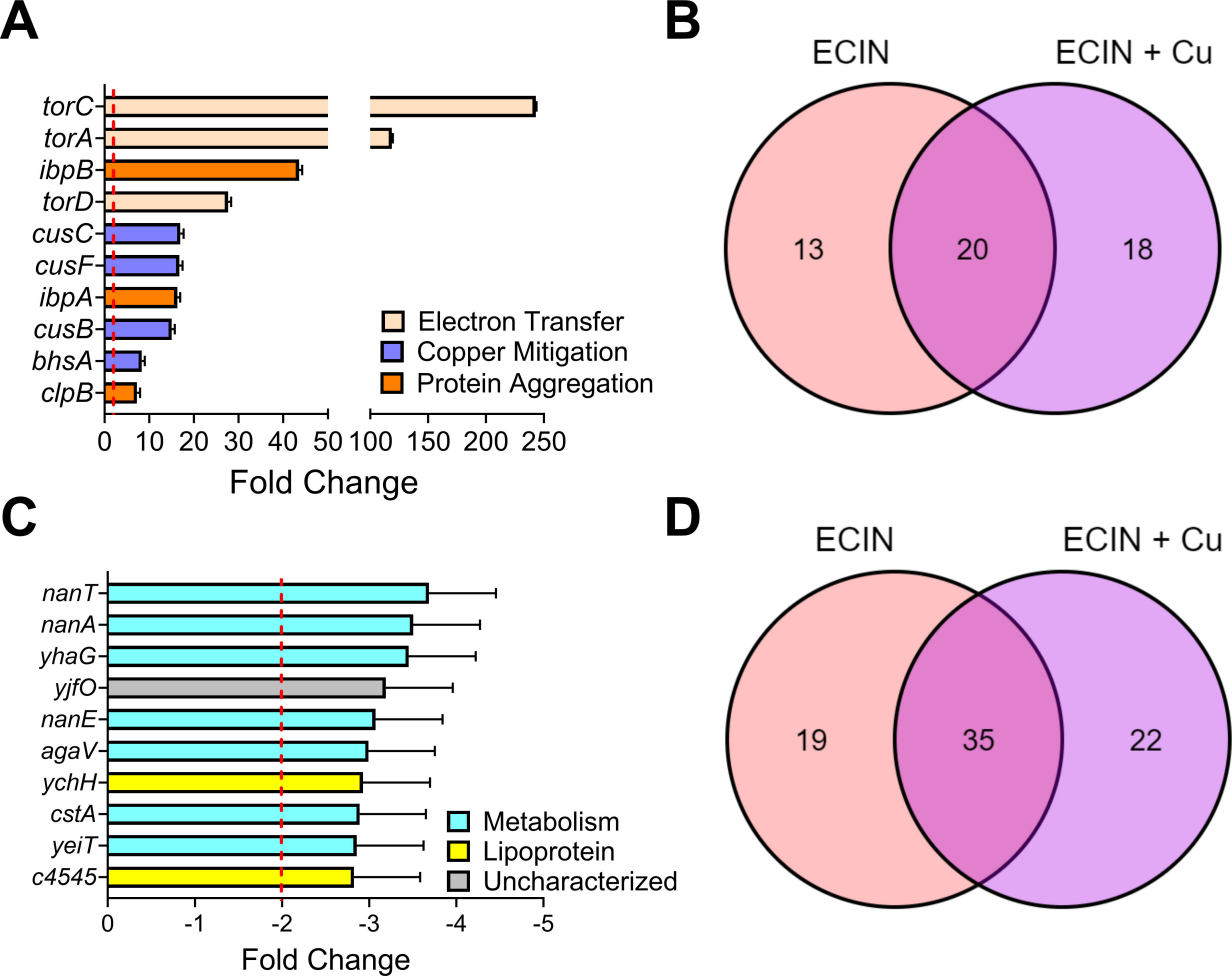
ECIN induces several stress response systems and halts metabolic processes. Representation of upregulated genes (**A and B**) and downregulated genes (**C and D**). RNAseq was performed on UPEC when treated with ECIN (1.3 µg/mL; 5 µM) at MIC and subinhibitory concentrations of copper (5 µM). The 10 most upregulated and downregulated genes are shown from UPEC treated with ECIN alone, and ECIN plus copper compared to the untreated control. Venn diagrams represent the number of unique or shared genes with other treatment groups between ECIN alone or ECIN plus copper. RNA was isolated three individual times and tested one time each (*N* = 3). Error bars represent SEM.

We performed transcriptional profiling to generate insights into the mechanism of action of ECIN. The transcriptional impact of ECIN plus copper, as well as ECIN and copper independently, on UPEC cultured in LB was analyzed via RNAseq and compared to untreated controls. The genes that were upregulated in different treatment groups are presented in Table S4 and Fig. 5A. When UPEC was treated with copper alone there was no significant change in the transcriptome. Treatment with ECIN in the absence or presence of copper upregulated a total of 33 and 38 genes, respectively (Fig. 5B; Table S4). The 10 most upregulated genes when UPEC was treated with ECIN plus copper are depicted in Fig. 5A. When comparing the upregulated genes, the combination of ECIN plus copper had 18 unique genes and shared 20 genes with the ECIN alone group (Fig. 5B; Table S4). The *torCAD* operon is among the most upregulated genes compared to the untreated control (Fig. 5A; Table S4). Regulatory genes including *marA* and *soxS*, orchestrating cellular responses to xenobiotic and superoxide stress, were upregulated by ECIN. UPEC exposed to ECIN appeared to experience envelope stress because *cpxP*, regulated by the CpxAR envelope stress response system, was induced. The *tor* operon is upregulated with or without the presence of copper (Table S4) and is reported to assist in the transport of electrons across the cell membrane during anaerobic respiration (37, 38). The genes *ipbA, ipbB, clpB, htpG,* and *htpX* are also among the top upregulated genes and are classically referred to as heat shock proteins associated with protein aggregation (39–41). Consistent with earlier reports on IpbAB and ClpB working together (39, 42), our results also identified increased abundance of transcripts of these genes in UPEC exposed to ECIN. We also noticed that multiple genes from the phage shock protein (*psp*) operon were upregulated in response to ECIN in UPEC (Table S4). Interestingly, ECIN also upregulates the *bhsA* (also known as *ycfR* or *comC*)*,* a gene reported to be involved in biofilm formation and mitigating copper stress (43, 44). The activity of *bhsA* is also associated with the regulation of the *cusCFBA* operon, the major copper-detoxification system in UPEC (30, 44, 45). Most importantly, the *cus* operon, a known copper-regulated efflux system, is upregulated in the presence of ECIN plus copper, but neither of them independently increased the expression of the *cus* genes (Table S4; Fig. 5A). Interestingly, ECIN triggers increased expression of multiple ribosomal genes, but this effect was abrogated when ECIN and copper were added in combination to the cultures (Table S4). Collectively, our results suggest that UPEC experiences enhanced copper stress and protein aggregation in the presence of ECIN.

### Expression of various metabolic pathways in UPEC is downregulated by ECIN

Our transcriptional profiling also revealed various metabolic pathways that are affected by the treatment of ECIN with or without copper (Fig. 5C; Table S5). The entire *nanATEKQ* operon, a sialic acid catabolic pathway, was downregulated in the presence of ECIN (Fig. 5C; Table S5) (46–48). Additionally, various genes associated with different sugar metabolic pathways were downregulated such as galactarate dehydratase (*yhaG*), pyruvate importer (*cstA*), dihydrothymine dehydrogenase (*yeiT*), and a potential mannose-binding PTS IIB protein (*agaV*) (49–53). Furthermore, *lacZ* encoding a beta galactosidase was also downregulated in the presence of ECIN. Collectively, these downregulated transcripts suggest that ECIN hinders multiple metabolic pathways in UPEC; however, further studies are needed to investigate whether these transcriptional changes lead to differences in metabolite levels.

### Supplementation of cysteine inhibits the activity of ECIN

**FIG 6:**
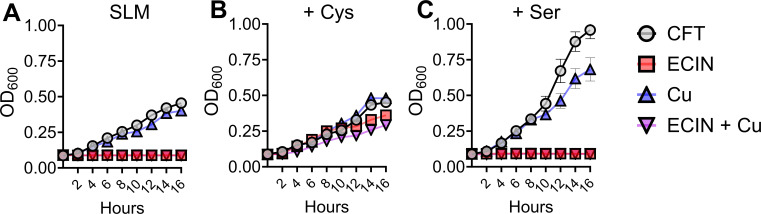
Cysteine rescues UPEC growth in the presence of ECIN. UPEC strain CFT073, was cultured in SLM in the presence or absence of ECIN (1.3 µg/mL; 5 µM), copper sulfate (5 µM), combination (5 µM each). Optical density was measured over time (*n* = 9). Error bars represent SEM. Cys, L-cysteine (1 mM) and Ser, L-serine (1 mM).

Isothiazolones are known to target cysteine (54–56). Our transcriptome analysis identified genes involved in cysteine metabolism (*cysD* and *cysP*). We investigated the interaction of cysteine with ECIN. First, we established the growth pattern of UPEC in sulfur-limiting medium (SLM) and compared that to the cysteine auxotroph *ΔcysE,* and thiosulfate import defective *ΔcysPUWA* mutants (Fig. 6; Fig. S7). As expected, the wild type and *ΔcysPUWA* mutant strains grew in SLM with and without copper (Fig. 6A; Fig. S7D). However, wild-type and mutant strains did not grow when treated with ECIN in the presence or absence of copper (Fig. 6A; Fig. S7A and D). The *ΔcysE* mutant did not grow in SLM regardless of copper or ECIN supplementation (Fig. S7A). When the media were supplemented with physiologically relevant concentrations of cysteine, growth of the wild type and mutant strains was rescued from inhibition by ECIN (Fig. 6B; Fig. S7B and E) (57). To determine whether this growth rescue was specific to cysteine, SLM was supplemented with serine, an amino acid identical to cysteine but with an oxygen atom instead of a sulfur atom. Serine supplementation displayed growth pattern identical to that of SLM, indicating the specificity of cysteine as an inhibitor of ECIN activity against UPEC (Fig. 6C; Fig. S7C and F).

### Evaluation of cytotoxicity of ECIN to HepG2 human hepatocyte cell line

**FIG 7:**
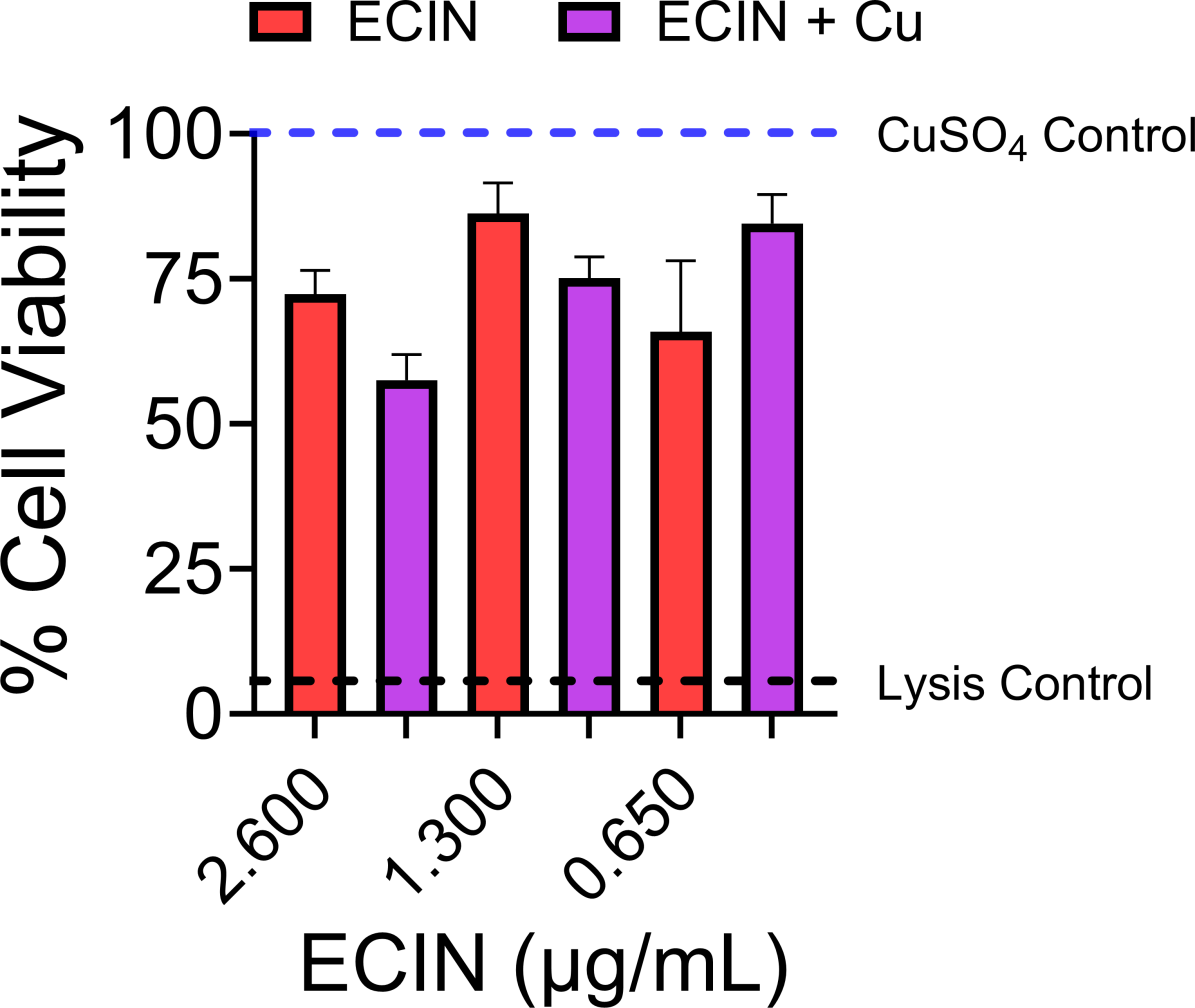
ECIN is weakly cytotoxic to a hepatic human cell line. ECIN in the presence or absence of copper was tested against the hepatic cell line HepG2 to determine cytotoxicity. Shades of red indicate cell viability in ECIN (2.5–10 µM) treated groups while shades of purple indicate the cell viability in groups treated with ECIN plus copper. No significant difference was found between treatments (*n* = 9, ANOVA with Bonferroni). Error bars represent SEM.

To begin evaluating the potential safety before testing ECIN *in vivo*, we determined the cytotoxicity of this molecule against HepG2 cells. Monolayers were exposed to various concentrations of ECIN or controls for 24 h before the addition of resazurin. The LD_50_ of the ECIN inferred from resazurin reduction was 3.47 and 4.27 µg/mL in the absence and presence of Cu, respectively. HepG2 cells treated with ECIN at 1× MIC (1.3 µg/mL; 5 µM) retain >75% viability (Fig. 7). These results indicate that ECIN can be used to inhibit bacterial growth without causing significant damage to eukaryotic cells in the presence or absence of copper.

### Interaction of ECIN with antimicrobials commonly used in the treatment of UTI

Given the increase in antimicrobial resistance against agents in clinical use against UTI, we tested whether ECIN could increase the efficacy of these agents to eradicate UPEC. Checkerboard assay conducted with ECIN and select antimicrobials against UPEC strain CFT073 revealed an additive interaction between ECIN and ciprofloxacin, fosfomycin, and nitrofurantoin (Table 2). However, the interaction between ECIN and trimethoprim sulfamethoxazole was indifferent against UPEC CFT073 (Table 2).

**TABLE 2:** Interaction of ECIN with antimicrobials commonly used against UTI^*a*^

Antibiotic	Mean FICI	SD	SEM	Interaction
Fosfomycin	0.515	0.000	0.000	Additive
Ciprofloxacin	0.785	0.000	0.000	Additive
Nitrofurantoin	0.897	0.016	0.009	Additive
TMP-SMZ	1.354	0.253	0.146	Indifferent

^*a*^ SD, standard deviation; SEM, standard error of the mean.

## DISCUSSION

The threat of antibiotic resistance is becoming a challenge to standard medical practices (12). Unfortunately, no new antibiotic families have been discovered since the late 1980s, and only a few new antibiotics have been used clinically since 2000 (12). Therefore, there is an urgent need to develop novel and alternative strategies to treat bacterial infections. Here, we set out to identify novel small molecules that inhibit the growth of UPEC, a common clinical indication for antibiotic use.

Host innate immune system sequesters or uses transition metals to impede pathogen growth (17–20). We and others have reported that copper is mobilized to the urinary tract during UTI as a host innate immune response to mitigate infection (16, 28–30, 58). Given the historic use of copper for antimicrobial applications combined with recent advances in understanding the contribution of copper to antimicrobial immunity, it is critical to identify and evaluate antimicrobials that work in concert with copper. The unique approach of our small molecule screen was to identify a copper-responsive inhibitor of UPEC because copper is an effector of nutritional immunity during UTI. Screens for copper-responsive inhibitors of *S. aureus* (59, 60) and *Mycobacterium tuberculosis* (61) have been reported. Gram-negative bacteria have been screened against iron uptake inhibitors, but copper-dependent high throughput screens have not been reported for Gram-negative pathogens (32, 62, 63). Here, we report the results of our small molecule screen that detected an isothiazolone, which we named ECIN, as a molecule of interest.

Isothiazolones are members of the chemical class of isothiazoles – a group of five heteroaromatic rings where a nitrogen atom occupies the second position (54). Some members of this class are used to treat anxiety and depression, but most of them are used in various commercial applications (54, 64–66). ECIN is a derivative of methylchloroisothiazolinone (MCIT), which is used as an industrial biocide (54, 67), and inhibits various bacteria (55, 68–72). Our results establish that ECIN acts as a bactericidal agent with a broad spectrum of activity (Table 1 and Fig. 3). Additionally, the MIC of the parental MCIT against *E. coli* is similar to the MIC of ECIN against *E. coli* and other pathogens determined in our study (Table 1) (68). It is remarkable that ECIN is highly effective against pathogens with inherently high antimicrobial resistance such as *P. aeruginosa* and *A. baumannii*, and antimicrobial-resistant strains such as methicillin-resistant *S. aureus* and UPEC isolates (HM16, HM60, and HM69). Our attempts to isolate ECIN-resistant UPEC by culturing high-density inocula (~10E11 CFU) on ECIN-containing LB agar or broth did not yield any resistant colonies. ECIN was supplemented at MIC levels or higher in experiments aimed at isolating resistant mutants. Ongoing studies in our group are directed at determining whether ECIN resistance could arise, and whether we could use those mutants to detect the mechanism of action of ECIN. ECIN is not cytotoxic against a human hepatocyte cell line at MIC levels (Fig. 7). In summary, ECIN acts as a broad-spectrum bactericidal agent that presents minimal cytotoxicity to hepatocyte cell line *in vitro*.

The antimicrobial activity of the isothiazolones is attributed to the cleavage of the N-S bond (54). The cleavage of this bond is due to the chalcogen bonding where the sulfur atom reacts with the nucleophilic components of cells (73). Isothiazolones are known to primarily target cysteine in active sites or in metabolites such as glutathione (54–56). An isothiazolone (benzisothiazolone) is reported to inhibit sortase A, a cysteine protease, and major virulence factor in *S. aureus* (74). Interestingly, our comparative transcriptome analysis revealed that *cysP* and *cysD* genes associated with thiosulfate uptake and cysteine metabolism were upregulated in UPEC exposed to ECIN in the presence of copper (Table S4). This finding suggests an increased cellular demand for cysteine during ECIN exposure. Our growth curve analysis in sulfur-limited media of wild-type, cysteine auxotroph (*ΔcysE*), and thiosulfate uptake mutants (*ΔcysPUWA*) further demonstrates the interplay between cysteine availability and bactericidal activity of ECIN (Fig. 6; Fig. S7). We are currently working toward identifying the molecular targets and mechanism of action of ECIN against UPEC.

Our comparative transcriptome analysis revealed that ECIN affects various stress response systems and downregulates several metabolic processes in the absence or presence of copper (Fig. 5; Tables S4 and S5). Most notably, our comparative transcriptomics revealed that in the presence of ECIN and subinhibitory copper, the *cus* system genes are upregulated in UPEC, which was not observed in copper-treated groups. This observation serves as a critical piece of data that further validate our results on the additive interaction between ECIN and copper. Our transcriptome analysis reveals that ECIN upregulates the expression of genes in the CpxR and MarA regulons. Genes in these pathways have been associated with resistance to multiple antimicrobial families including beta-lactams, aminoglycosides, chloramphenicol, quinolones, and tetracyclines (75–79). Furthermore, our findings on ECIN-responsive transcriptome in UPEC identified multiple genes that were previously reported as differentially expressed in *E. coli* during copper stress (80, 81). Select genes in this category include *cusC* and *copA*, involved in copper efflux; *cpxP*, a membrane stress response gene; *soxS,* a superoxide stress response gene; and *htpX*, degradation of unstable membrane proteins gene (80). It is important to note that ECIN alone or in combination with copper induces the expression of these genes in UPEC in this study reinforcing our results that ECIN augments copper stress. Additionally, fluoroquinolone-resistant UPEC isolates are known to express the *psp* genes at a higher level compared to fluoroquinolone-sensitive UPEC isolates (82). Here, we report that *pspA* and *pspD* genes are upregulated when treated with ECIN with or without copper. Our findings, taken together with results on transcriptional changes in *E. coli* in response to antimicrobials and copper, indicate that ECIN activates multiple pathways involved in promoting bacterial survival during stress imposed by antimicrobials and copper. Our findings on ECIN-responsive transcriptome are consistent with previous reports on copper stress as a promoter of protein aggregation in *E. coli* (83). We are currently following up on other differentially expressed genes detected in our study to gain insights into the mechanism of action of ECIN. Collectively, our data support a model, in which ECIN acts as a copper-responsive molecule to inhibit UPEC.

We and others have reported on the highly interconnected nature of iron and copper homeostasis systems in UPEC (45, 58, 84). High throughput screens incorporating iron limitation, a facet of nutritional immunity, to detect antimicrobials against UPEC and *P. aeruginosa* have been reported (32, 62, 63). Our results reveal that ECIN does not impact iron availability in UPEC, and inhibition of bacterial growth appears to be independent of iron availability. In contrast with UPEC inhibitors of iron uptake that act as bacteriostatic agents (32), ECIN exhibits bactericidal activity. Our growth curve and viability analyses reveal that ECIN is bactericidal, and comparable to that of the control for bactericidal activity, ciprofloxacin, a widely used bactericidal antibiotic in the clinical management of UTI. ECIN is also bactericidal against multiple UPEC strains including multidrug-resistant isolates (Fig. 3; Fig. S6).

Our comparative transcriptome analysis also revealed multiple genes associated with either development or maintenance of biofilms (Fig. 5; Tables S4 and S5) (43, 85–87). Biofilm formation is a major virulence factor that shields the bacteria from the host immune response and often leads to the failure of antimicrobial therapy (3, 88). Our results demonstrate that ECIN is highly effective in preventing UPEC biofilm growth, even at 0.5× MIC (Fig. 4A). However, ECIN did not eradicate preformed biofilms *in vitro* (Fig. 4B). These findings imply that ECIN is less likely to clear established biofilms on catheters, which are often found in patients with complicated UTI.

The concentration of copper used in our small molecule screen is aligned with the levels found in blood and is higher than that in urine during UTI (28). This was used to account for any sequestration of the supplemented copper by the media while maintaining physiological relevance (31). We used crystal violet as an indicator for biofilm biomass. Although it is a commonly used technique, it does not capture the viability and metabolic activity of UPEC biofilms in the presence of ECIN, especially when subinhibitory concentrations of ECIN allow UPEC growth but completely inhibit biofilm formation (Fig. 4). Our ICP-MS analysis reveals cell-associated copper content, but it does not distinguish the cellular compartment where copper is localized. As ECIN upregulates the expression of *cus* genes, it is highly likely that copper is localized to the periplasm during ECIN exposure. Notwithstanding these limitations, our study detects and characterizes a novel copper-responsive inhibitor of UPEC and a broad range of uropathogens.

Overall, here, we report the detection and characterization of a potential new antimicrobial agent that works additively with copper, an effector of nutritional immunity, and is effective against uropathogens that cause UTI, a leading global public health problem. Future directions for our work include defining the mechanism of action and evaluating the safety and efficacy of ECIN in a murine model of UTI.

## MATERIALS AND METHODS

### Bacterial strains and culture conditions

Bacterial strains used in this study are listed in Table S1. Experiments were performed with UPEC strain CFT073, a prototypical pyelonephritis strain, unless otherwise stated. Overnight cultures of bacteria were grown in lysogeny broth (LB Lennox, Fisher Bioreagents) shaking at 200 rpm in 37°C overnight. Overnight cultures were adjusted to an OD_600_ = 1.0 and diluted 1:100 in either LB, Mueller-Hinton Broth (MHB, BD), Tryptic Soy Broth/Agar (TSB/TSA, BD), filter-sterilized female donor urine with no history of UTI (Cone Bioproducts), YESCA medium (89, 90), M9 minimal medium (M9MM), or sulfur-limited medium (91) for various assays.

### The small molecule library

The small molecule library in the Sacchettini Laboratory was used in our screening assays. This library comprised 51,098 unique small molecules and was assembled by randomly selecting compounds with a high structural diversity and medicinal properties. Details on the construction and characteristics of the screening library have been previously described (92, 93). Each compound is dissolved to ~1 mM in DMSO using an average molecular weight of 250 g/mol for the entire library.

### High throughput screening: primary screening

Glycerol stocks of UPEC strain CFT073 Δ*tolC* were generated, aliquoted, and stored at −80°C. Bacteria were then sub-cultured 1:100 into LB supplemented with 25 µM CuSO_4_. Using an Analytik Cybio liquid handling machine, 24 µL of copper-supplemented LB was added to 384 well plates (Corning Cat# 3765). 1 µL from the small molecule library was then added to the wells of the screening plate. Next, 25 µL of bacteria in the copper-supplemented LB were added to every well. Finally, 10 µL of 0.02% resazurin was added to each well to determine the metabolic activity after 24 h. Each well had a total volume of 60 µL with an approximate drug concentration of 16.7 µM. The compound ethyl 3-[(1e)−3,3-dimethyl-1-triazenyl]−1h-indole-2-carboxylate was used as the positive control for bactericidal activity (16.7 µM, or diluted from 16.7 to 0.13 µM; ChemSpider ID #17736645). Vehicle controls containing DMSO were also used. The screening plates were incubated at 37°C for 24 h. The fluorescence intensity values (Ex 540 nm and Em 590 nm) were measured using a FLUOstar Omega plate reader (BMG Labtech) with 50% gain intensity adjustment. Data were then uploaded to the Collaborative Drug Discovery (CDD) portal (www.collaborativedrug.com), and Z-scores were calculated in comparison to the control wells for each of the individual plates. The fluorescence values of test wells with a Z-score ≤ −3.5 were declared a hit.

### High throughput screening: secondary screening

Hits from the primary screen were evaluated for copper-dependent activity. Compounds were serially diluted in a 1:1.4 ratio (16.7–0.81 µM) and incubated with UPEC in the presence or absence of 25 µM CuSO_4_ at 37°C for 24 h. The 50% growth IC_50_ was calculated by using the curve fit four-parameter logistic function $\text{y = bottom +}\frac{\text{top-bottom}}{1+10^{(\log_{{\text{IC}}_{50}}-\;\log_{\text{X}})\text{* hillslope}}}$, where y is the measured concentration of the hits, top indicates the negative control (no inhibitor; 100% growth), and bottom indicates the positive control (full inhibition; 0% growth). A decrease in IC_50_ of ≤1.0 µM in the presence of copper was selected as a copper-responsive hit.

### High throughput screening: additional screenings

The dose-response curves were performed of copper-responsive hits (tertiary screening), and visually evaluated to assess their activity and relevance. Compounds that produced a sigmoidal-shaped dose-response curve were determined to be a viable, non-toxic, and copper-responsive compound. Procedures for the secondary and tertiary screening were repeated against the CFT073 wildtype and Δ*tolC*, to evaluate hit specificity of the wildtype and mutant (quaternary screening). Plates were incubated at 37°C for 24 h.

### MICs

Overnight cultures of bacteria were grown in LB for uropathogens, except *S. aureus*, which was cultured in TSB. Bacterial cultures were then adjusted to an OD_600_ = 1.0 and diluted 1:100 in fresh media. Serial twofold dilutions of ECIN from 10.4 µg/mL to 39.0 ng/mL were performed. Medium-alone and no ECIN controls were included on each plate. Assay plates were allowed to incubate for 16 h at 37°C. MIC was visually determined and recorded. Each bacterium was tested at least three separate times with three technical replicates (*n* = 9). The MIC is the median of all replicates.

### Kill curves

Overnight cultures of UPEC were diluted 1:100 in fresh LB containing 0, 25, and 250 µM of CuSO_4_. ECIN was diluted 1:1.4 in a master plate at 50× of final concentration and transferred to the assay plates. Kill curves were performed three separate times with three technical replicates (*n* = 9). IC_50_ was calculated with the four-parameter nonlinear regression test in GraphPad Prism 10.

### Checkerboard assays

Bacterial cultures were challenged with ECIN and CuSO_4_ to determine whether the two worked synergistically, additively, or antagonistically. Figure S2A shows the plate layout used in this experiment. ECIN and CuSO_4_ were added at 2×/4× levels to row A and column 1, respectively. Next, row-wise followed by column-wise serial dilutions were performed (Fig. S2B). Mock dilutions of either ECIN (Fig. S2C) or CuSO_4_ (Fig. S2D) were performed as well. The FICI was calculated as $\frac{MIC\;of\;antibiotic\;A\;alone}{MIC\;of\;antibiotic\;A\;in\;combination}+\frac{MIC\;of\;antibiotic\;B\;alone}{MIC\;of\;antibiotic\;B\;in\;combination}$. The FICI was calculated from each row passing the MIC baseline and averaged together to determine the overall FICI score for the plate. The assay was repeated three separate times. The FICI scores of <0.5, 0.5–1, 1–4, > 4 was used to define synergistic, additive, indifferent, and antagonistic activities between ECIN and CuSO_4_ and CuCl_2_ (34, 35). Additionally, we tested the interaction ECIN has with antibiotics with clinical relevance to UTI against CFT073. The MIC for the antibiotics used were: ciprofloxacin (32 ng/mL), fosfomycin (2 µg/mL), nitrofurantoin (32 µg/mL), and trimethoprim-sulfamethoxazole (500 ng/mL).

### Zone of inhibition assays

The copper-responsive effect ECIN on various pathogens was evaluated via zone of inhibition assays. Adjusted bacterial cultures (OD_600_ = 1) were spread on LB agar (200 µL). Blank paper discs (Remel) were placed on the agar, 5 µL of 100 mM ECIN was added to the paper discs and incubated for 24 h before recording zones of inhibition. *P. mirabilis* and *S. aureu*s were plated on LB for an equal comparison to other uropathogens. Additionally, *P. mirablils* was also tested in LB agar without NaCl to minimize the effects of swarming. *S. aureus* was also tested on tryptic soy agar for optimal growth.

### RNA isolation

UPEC strain CFT073 in mid-log phase (2 h after subculture) was treated with 1.3 µg/mL ECIN, 5 µM CuSO_4_, or ECIN and CuSO_4_ in combination for 30 min. RNA was stabilized with RNA Protect, and isolated with RNeasy Mini Kit (Qiagen) and contaminating DNA was removed with DNase (Invitrogen). RNA, prepared as described here from three independent experiments, was used for qPCR and RNAseq.

### Quantitative PCR

cDNA libraries were synthesized with Superscript III reverse transcriptase (Invitrogen). qPCR was performed with SYBR Green (Thermo Scientific) in a Bio-Rad CFX Real-Time System with the oligonucleotide primers listed in Table S2. The housekeeping gene *gapA* was utilized to normalize transcript levels. Relative expression was evaluated by ΔΔCT method using untreated controls of UPEC strain CFT073 as baseline (94).

### Differential expression via comparative transcriptomics

RNA quality was assessed in a Tapestation system (Agilent). A strand-specific library was prepared with NEBNext Ultra II Directional RNA Library Prep after depleting bacterial rRNA. Paired-end Illumina data (2 × 300 bp) generated at Texas A&M Genomics Core facility was processed in Galaxy (Bowtie2 and FeatureCounts) and differential expression was determined with DeSeq2 in R. Genes with an FDR-corrected *P* value ≤ 0.05 with a fold change of in expression >2/<−2 were selected as differentially expressed genes. Raw reads can be accessed in SRA PRJNA1079753. Venn diagrams of differentially expressed genes were created with https://molbiotools.com/listcompare.php.

### ICP-MS

Cell-associated levels of copper, manganese, iron, and zinc were measured by ICP-MS in UPEC cultures (mid-log phase) exposed to 1.3 µg/mL ECIN, 5 µM CuSO_4_, or ECIN and CuSO_4_ in combination for 30 min, as described previously in Methods and Materials: RNA Isolation. Samples were pelleted, washed with 10 mM HEPES, and digested with trace element grade HNO_3_ (Fisher Scientific). Operators were blinded to sample identity during ICP-MS analysis. ICP-MS was performed in single quadrupole mode with helium gas in the collision/reaction to minimize potential spectral interferences (8800 ICP-MS/MS, Agilent Technologies).

### Biofilm assays

The UPEC strain UTI89 was grown overnight in LB and adjusted to an OD_600_ = 1.0. Cultures were diluted 1:100 in MHB with or without indicated concentrations of ECIN and incubated for 24 h at 30°C in 96-well polystyrene plates. The next day, bacterial growth was measured by obtaining the OD_600_. The medium and planktonic cells were then removed, biofilms were stained with 0.1% crystal violet, which was dissolved in acetic acid before determining OD_550_ (36). Biofilm biomass was normalized to the bacterial density $\left(\frac{\text{O}{\text{D}}_{\text{550}}}{{\text{OD}}_{\text{600}}}\right)$. For assessing eradication, biofilms were allowed to grow and establish for 24 h in YESCA. After 24 h, the spent medium was removed and replaced with fresh YESCA and treated with ECIN for an additional 24 h.

### Cysteine assays

UPEC was grown overnight in LB and centrifuged at 4,000 RPM for 10 min. The supernatant was discarded, and the pellet was resuspended in sulfur-limited media (SLM) previously described and adjusted to an OD_600_ = 1.0 (91). SLM contained 40 mM K_2_HPO_4_, 15 mM NaH_2_PO_4_, 19 mM NH_4_Cl, 90 µM CaCl_2_, 3 mm MgCl_2_, 50 µM MgSO_4_, and 30 mM glucose (91). UPEC was then grown for 16 h in SLM or SLM supplemented with either 1 mM cysteine or 1 mM serine to mimic the concentration of cysteine found in human urine (57). Two mutants, Δ*cysE* (a cysteine auxotroph) and Δ*cysPUWA* (a thiosulfate import) were used as controls as done previously (30). The assay was performed three separate times with three technical replicates.

### Cytotoxicity assays

Human hepatocyte cell line HepG2 (ATCC HB-8065) was grown in Eagle’s Minimum Essential Medium (EMEM; ATCC #30–2003). Cells were seeded in black, clear bottom 96-well plates (Thermo Scientific) with a seeding density of 5.5 × 10^4^ cells per well. Cells were grown to ~70% confluency and then treated with various concentrations of ECIN in the presence or absence of 5 µM CuSO_4_. After 24 h, 0.02 mg/mL resazurin was added to each well and incubated for 4 h. Endpoint fluorescent activity (Ex 540 nm and Em 590 nm) was measured as the reduction of resazurin to resorufin, an indicator for metabolic activity. The final concentration of DMSO in each well was 0.0032% to minimize the toxicity of DMSO. Controls included on this plate included a vehicle control (DMSO), a CuSO_4_ (5 µM) control, a complete lysis control (1% Triton 100×), and an untreated control. Cytotoxicity of HepG2 cells was calculated by determining the cell viability of each well normalized to the vehicle alone control ($\frac{\text{read value}}{\text{average vehicle control}}*100\%$).

### Statistical analysis

Results were analyzed with statistical tests indicated throughout Materials and Methods in GraphPad Prism, and *P* < 0.05 was considered as a statistically significant difference.

## SUPPLEMENTAL MATERIAL

Supplemental material jb.00112-24-s0001.pdf Tables S1 to S5; Fig. S1 to S7.

ASM does not own the copyrights to Supplemental Material that may be linked to, or accessed through, an article. The authors have granted ASM a non-exclusive, world-wide license to publish the Supplemental Material files. Please contact the corresponding author directly for reuse.
