# Supplementary material for: Identification of a copper-responsive small molecule inhibitor of uropathogenic Escherichia coli
Source: J Bacteriol. 2024 Jun 10;206(7):e00112-24. doi: 10.1128/jb.00112-24 (PMC11270900; doi:10.1128/jb.00112-24)
Supplement: Supplemental material — Tables S1 to S5; Fig. S1 to S7. [file jb.00112-24-s0001.pdf]

Table S1. Strains utilized in this study.

Bacteria	Strain	References
E. coli	CFT073	(1-7)
	CFT073 Δ*tolC*	(8)
	CFT073 Δ*cysE*	(9)
	CFT073 Δ*cysPUWA*	(9)
	CFT073 ΔΔcusRSCFBAcopA	(10)
	UTI89	(4, 11)
	HM16	(9)
	HM60	(9)
	HM69	(9)
	ABU83972	(12)
K. pneumoniae	ATCC 43816	(13-17)
P. mirabilis	HI4320	(18)
S. marcescens	WF191	(3)
A. baumannii	ATCC 17978	(19-21)
P. aeruginosa	ATCC 27312	(22, 23)
	ATCC 27583	(23-25)
	ATCC 49189	(26-28)
S. aureus	SF8300, USA 300	(6, 29)
	SA116, USA 300	(6, 30)

Table S2. Oligonucleotide primers utilized in this study.

Primer ID[a]	Sequence (5’-3’)
gapA F	AAGTTGGTGTTGACGTTGTCGC
gapA R	AGCGCCTTTAACGAACATCG
cusC F	AATGTCGCGCAAAGCTATTT
cusC R	CGACAAACGCATATGACTGC

[a]F, forward; R, reverse

Table S3. MIC of ECIN vs CFT073 in various media in the presence or absence of Cu.

Medium	Cu (+)		Cu (-)	
	µg/mL	µM	µg/mL	µM
LB	1.300	5.000	1.300	5.000
MHB	1.300	5.000	1.300	5.000
TSB	1.300	5.000	1.300	5.000
Urine	1.300	5.000	1.300	5.000
M9MM	0.163	0.625	0.325	1.250
SLM	0.325	1.250	0.650	2.500

LB, Lysogeny Broth; MHB, Mueller-Hinton Broth; TSB, Tryptic Soy Broth; M9MM, M9 Minimal Media; SLM, Sulfur-Limited Media

Table S4. UPEC genes upregulated in the presence of ECIN

Genes Upregulated by ECIN:		Fold Change:
torC	Electron Transport	206.139
torA	Electron Transport	109.297
torD	Electron Transport	26.592
ibpB	Protein Aggregation	12.946
ibpA	Protein Aggregation	11.028
clpB	Protein Aggregation	3.672
marA	Cytoplasmic Stress	3.631
yncJ	Hypothetical Protein	3.315
bhsA	Biofilm Formation/Copper Stress	3.007
yjbO	Hypothetical Protein	2.916
marR	Cytoplasmic Stress	2.902
soxS	Oxidative Stress	2.579
marB	Cytoplasmic Stress	2.348
rrsB	16s Ribosomal Subunit	2.309
rrsA	16s Ribosomal Subunit	2.285
cpxP	Membrane/Envelope Stress	2.274
rrsE	16s Ribosomal Subunit	2.255
rrsH	16s Ribosomal Subunit	2.246
rrsD	16s Ribosomal Subunit	2.218
c0368	Hypothetical Protein	2.216
rrsC	16s Ribosomal Subunit	2.193
malZ	Maltodextrin Glucosidase	2.189
mutM	DNA Glycosylase	2.163
rrsG	16s Ribosomal Subunit	2.160
c0778	Hypothetical Protein	2.156
pspA	Membrane Stress	2.119
ykgO	50s Ribosomal Subunit	2.116
rrlA	23s Ribosomal Subunit	2.106
yebE	Inner Membrane Protein	2.101
speF	Ornithine Decarboxylase	2.086
asnA	Asparagine Synthetase	2.080
yceO	Biofilm Formation / Acid Stress	2.073
pspC	Membrane Stress	2.037

Genes Upregulated by ECIN + Cu:		Fold Change:
torC	Electron Transport	243.033
torA	Electron Transport	118.868
ibpB	Protein Aggregation	43.560

torD	Electron Transport	27.660
cusC	Copper Stress	16.959
cusX	Copper Stress	16.730
ibpA	Protein Aggregation	16.270
cusB	Copper Stress	15.017
bhsA	Biofilm Formation / Copper Stress	8.340
clpB	Protein Aggregation	7.259
cusA	Copper Stress	6.295
marA	Cytoplasmic Stress	5.987
yncJ	Hypothetical Protein	5.346
soxS	Oxidative Stress	3.797
pspC	Membrane Stress	3.683
marB	Cytoplasmic Stress	3.504
ybeD	Type II Secretion System	3.494
pspA	Membrane Stress	3.429
yjbO	Hypothetical Protein	3.353
yebE	Inner Membrane Protein	3.277
marR	Cytoplasmic Stress	3.269
pspD	Membrane Stress	3.163
cpxP	Membrane/Envelope Stress	3.079
htpG	Protein Aggregation	2.712
asnB	Asparagine Synthetase	2.534
asnA	Asparagine Synthetase	2.459
yhdV	Lipoprotein	2.250
cysD	Sulfate Adenylyltransferase	2.205
htpX	Protein Aggregation	2.169
yjaI	Zinc Responsive Envelope Stress	2.143
malZ	Maltodextrin Glucosidase	2.084
cysP	Thiosulfate Uptake	2.075
alx	Membrane Redox Modulator	2.072
mutM	DNA Glycosylase	2.060
copA	Copper Stress	2.048
c1016	Hypothetical Protein	2.014
cspG	Cold Shock Protein	2.012
yciF	Osmotic Stress	2.003

Table S5. UPEC genes downregulated in the presence of ECIN

Genes Downregulated by ECIN:		Fold Change:
nanT	Sialic Acid Metabolism	3.370
nanA	Sialic Acid Metabolism	3.365
cstA	Pyruvate Transporter	2.834
nanE	Sialic Acid Metabolism	2.812
yhaG	Galactarate Dehydratase	2.584
nanQ	Sialic Acid Metabolism	2.513
yeiT	Dihydropyrimidine Dehydrogenase	2.490
nanK	Sialic Acid Metabolism	2.469
ycgC	Dihydroxyacetone Kinase	2.441
dhaK	Dihydroxyacetone Kinase	2.433
yeiA	Dihydropyrimidine Dehydrogenase	2.423
ydfZ	Hypothetical Protein	2.391
c2681	Hypothetical Protein	2.384
agaV	PTS IIB protein	2.334
ycgS	Dihydroxyacetone Kinase	2.334
ychH	Biofilm Formation / Membrane Stress	2.330
cadA	Lysine Decarboxylase	2.314
c3889	PTS IIB protein	2.280
melR	DNA Transcriptional Regulator	2.269
glpF	Glycerol Transporter	2.258
c4779	Hypothetical Protein	2.251
c4545	Hypothetical Protein	2.235
yjfN	Protease Activator	2.227
c1959	PTS IIB protein	2.203
ugpB	Periplasmic ABC Transporter	2.200
c4195	Hypothetical Protein	2.193
atoS	Sensor Histidine Kinase	2.184
yejG	Hypothetical Protein	2.180
glpK	Glycerol Kinase	2.177
ygfK	Fe-S Oxidoreductase	2.166
c4276	Hypothetical Protein	2.160
nmpC	Outer Membrane Porin	2.155
c0756	Hypothetical Protein	2.146
lacZ	Beta Galactosidase	2.145
ygfU	Urate Transporter	2.144
glcC	Glycolate Transcriptional Regulator	2.143
lacY	Lactose Permease	2.116
c1176	Hypothetical Protein	2.110

c2348	Outer Membrane Porin	2.103
c4205	Hypothetical Protein	2.101
c1166	Hypothetical Protein	2.096
c3890	PTS IIB protein	2.094
ydcH	Hypothetical Protein	2.079
ftnB	Ferritin-like Protein	2.069
yhiI	Putative ABC Transporter	2.056
agaS	Galactosamine Phosphate Isomerase	2.048
agaY	PTS IIB protein	2.045
c4288	Hypothetical Protein	2.040
c4493	Hypothetical Protein	2.031
ytfK	Hypothetical Protein	2.029
yjaI	Zinc Responsive Envelope Stress	2.014
nagE	PTS II protein	2.012
c4778	Hypothetical Protein	2.006
c4776	Hypothetical Protein	2.001

**Genes Downregulated by ECIN + Cu:**		**Fold Change:**
nanT	Sialic Acid Metabolism	3.686
nanA	Sialic Acid Metabolism	3.505
yhaG	Galactarate Dehydratase	3.454
yjfO	Biofilm Stress / Motility	3.192
nanE	Sialic Acid Metabolism	3.074
agaV	PTS IIB protein	2.994
ychH	Biofilm Formation / Cell Stress	2.930
cstA	Pyruvate Transporter	2.892
yeiT	Dihydropyrimidine Dehydrogenase	2.857
c4545	Hypothetical Protein	2.827
ycgS	Dihydroxyacetone Kinase	2.788
agaY	PTS IIB protein	2.737
yeiA	Dihydropyrimidine Dehydrogenase	2.720
melR	DNA Transcriptional Regulator	2.709
ugpB	Periplasmic ABC Transporter	2.705
nanK	Sialic Acid Metabolism	2.693
c1176	Hypothetical Protein	2.686
c4779	Hypothetical Protein	2.662
c3889	PTS IIB protein	2.634
yahN	Inner Membrane Serine Transporter	2.616
ycgC	Dihydroxyacetone Kinase	2.614
yjfN	Protease Activator	2.607
dhaK	Dihydroxyacetone Kinase	2.570

c4494	Hypothetical Protein	2.551
c1565	Prophage TonB Protein	2.542
atoS	Sensor Histidine Kinase	2.540
agaZ	Putatative Tagatose Aldolase Chaperone	2.537
c4775	Hypothetical Protein	2.529
c4276	Hypothetical Protein	2.527
ydfZ	Hypothetical Protein	2.524
yhiI	Putative ABC Transporter	2.511
ytfQ	Galactofuranose Transporter	2.502
c2681	Hypothetical Protein	2.486
glcC	Glycolate Transcriptional Regulator	2.479
nagE	PTS II protein	2.463
fadB	Fatty Acid Degradation	2.454
glaH	Glutarate Dioxygenase	2.447
c3271	Hypothetical Protein	2.440
nanQ	Sialic Acid Metabolism	2.440
trhA	Transmembrane Homeostasis	2.437
c4778	Hypothetical Protein	2.407
lacZ	Beta Galactosidase	2.383
nupG	Nucleoside Transporter	2.381
c4277	PTS EIIA Protein	2.377
c3890	PTS EIID Protein	2.364
agaS	Galactosamine Phosphate Isomerase	2.338
c4546	Hypothetical Protein	2.327
c4777	Deoxyphosphogalactonate Aldolase	2.326
ygjL	Fatty Acid CoA Reductase	2.301
agp	Periplasmic Glucose Phosphotase	2.282
yfcH	DNA Epimerase	2.260
caiF	Carnitine Metabolism	2.248
nmpC	Outer Membrane Porin	2.229
c4495	Hexuronate Transporter	2.165
ybhQ	Hypothetical Protein	2.162
acs	AMP Forming Acetyl-CoA Synthetase	2.097
c2348	Outer Membrane Porin	2.077

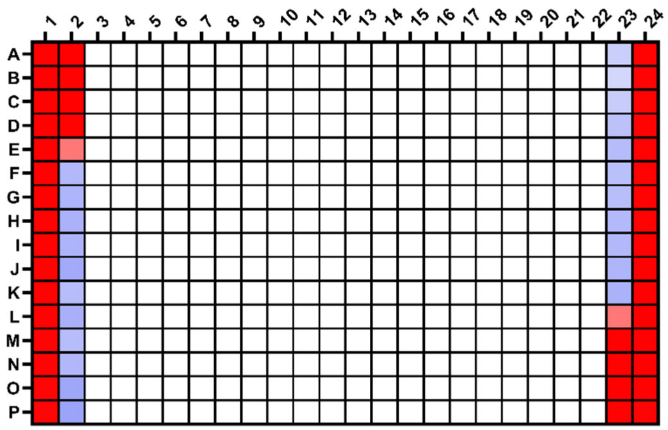

Figure S1. Map of primary screening assay. Columns 1, 2, 23, and 24 were used for controls. Red indicates positive control wells (no bacterial growth) and blue indicates negative control wells (bacterial growth). Columns 3 through 22 were used for screening compounds of the small molecule library.

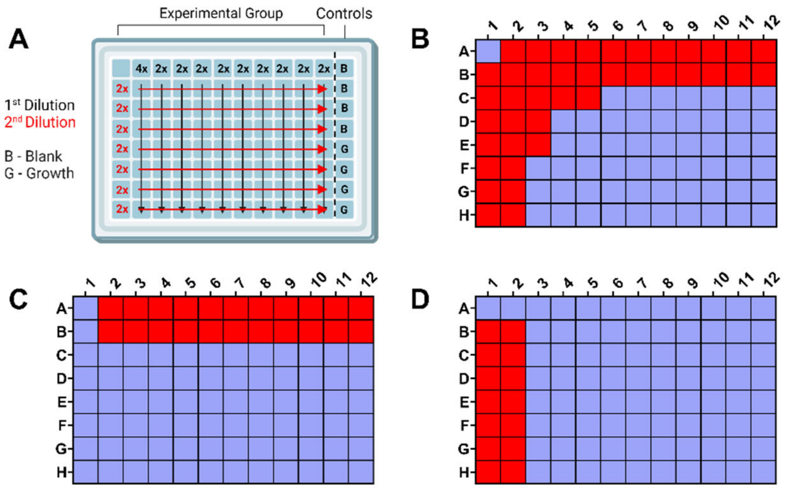

Figure S2. Layout of checkerboard assays. A. ECIN and copper were added to a 96 well plate at 4x / 2x MIC as indicated. ECIN was diluted along the columns. Copper was diluted across rows B = blank media alone without bacteria. G = untreated bacteria (n = 9). **B-D**. Results from a representative checkerboard assay with red indicating no turbidity and blue indicating turbidity. **B**, ECIN and copper. **C**. ECIN alone. **D**. Copper alone.

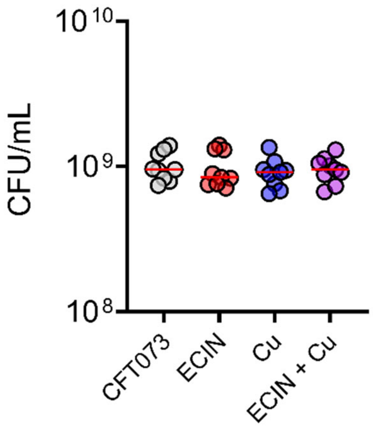

Figure S3. Acute exposure does not kill UPEC. Bacteria in mid-log phase were treated with ECIN at MIC (1.3 µg/mL; 5 µM) in the presence or absence of sub-inhibitory levels of copper (5 µM) for 30 minutes. Line represents median. Experiments were repeated three separate times with three technical replicates each (n = 9). No significant difference was detected (Kruskal-Wallis test).

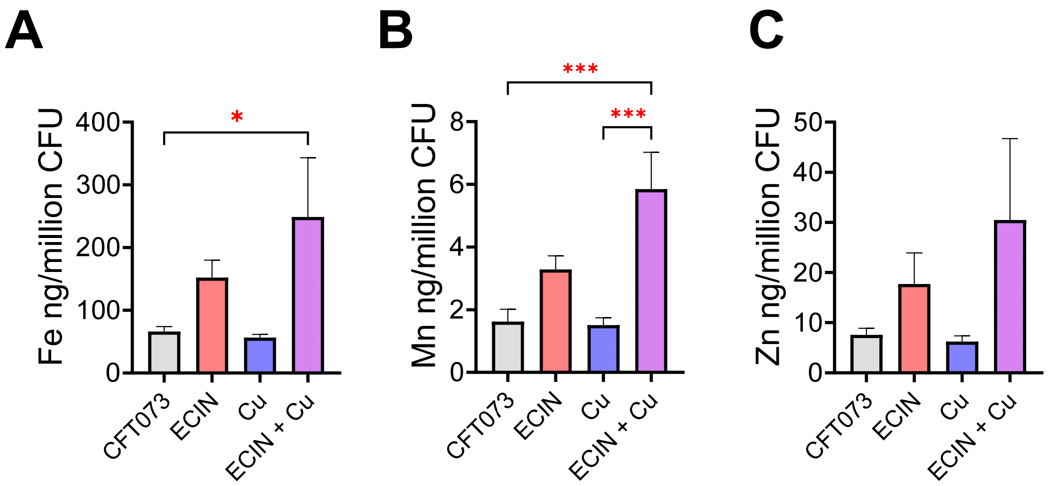

Figure S4. Changes in cellular levels of other transition metals involved in nutritional immunity. ICP-MS revealed that the combination of ECIN (1.3 µg/mL; 5 µM) plus copper (5 µM) elevates the amount of cell-associated iron (**A**), manganese (**B**), and zinc (**C**) in UPEC (n = 9). ANOVA with Bonferroni, **P* < 0.05, and ****P* < 0.001.

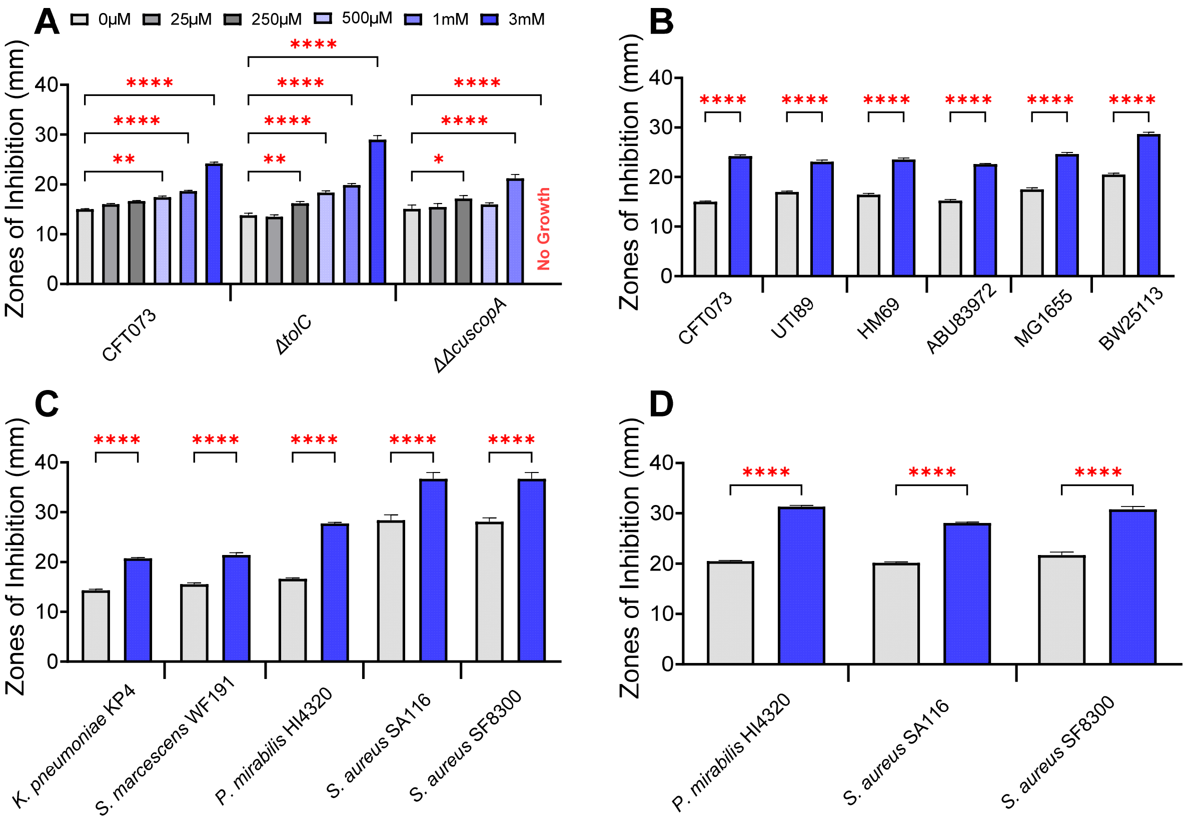

Figure S5. ECIN has a copper-dependent broad-spectrum effect on various bacteria. Zone of inhibition assays were utilized to determine the copper-dependent inhibition of ECIN in LB agar supplemented with various concentrations of $CuSO_4$ (A-C). **A.** ECIN vs *E. coli* strain CFT073 wildtype and mutants lacking *tolC* and *cusSRCFBAcopA* genes. **B**. ECIN vs various *E. coli* strains. UPEC: CFT073, UTI89, HM69; Asymptomatic bacteriuria: ABU 83972, commensal-derived lab strains: MG1655 and BW25113. **C.** ECIN vs other uropathogens. **D**. Zones of inhibition for ECIN vs *P.* mirabilis in LB agar containing no NaCl, and for *S. aureus* grown on TSA. N = 9. ANOVA with Bonferroni*$P < 0.05$, **$P < 0.01$, and ****$P < 0.0001$.

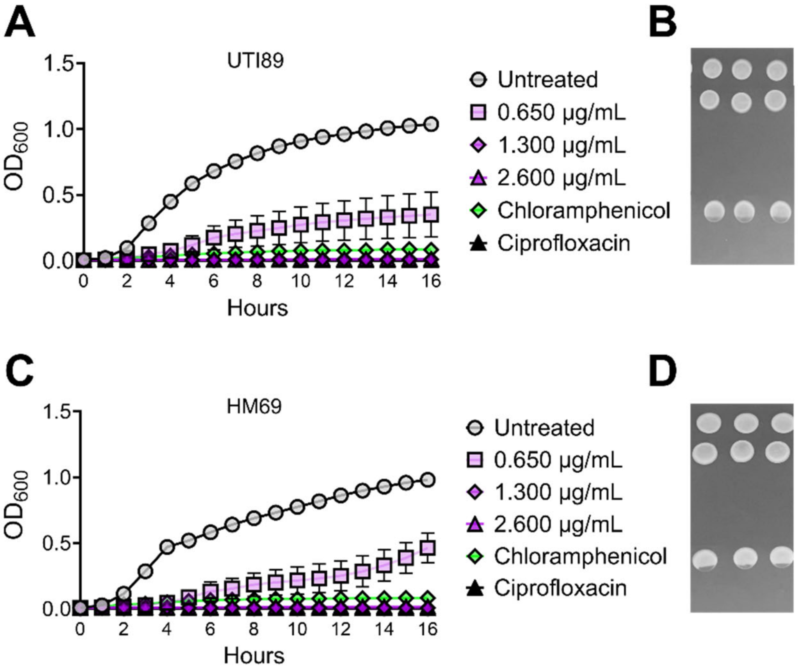

Figure S6. ECIN is bactericidal against UPEC strains UTI89 and HM69. A & C. Optical density of UPEC UTI89 and HM69 cultures containing ECIN (1.3 μg/mL; 5 μM) or controls over time. Chloramphenicol (MIC = 8 μg/mL) and ciprofloxacin (MIC = 32 ng/mL) were used as bacteriostatic and bactericidal controls, respectively (n = 9). Error bars represent SEM. **B & D.** The cultures used for generating the growth curves were spot plated on LB agar to determine the presence of viable bacteria from UTI89 and HM69, respectively.

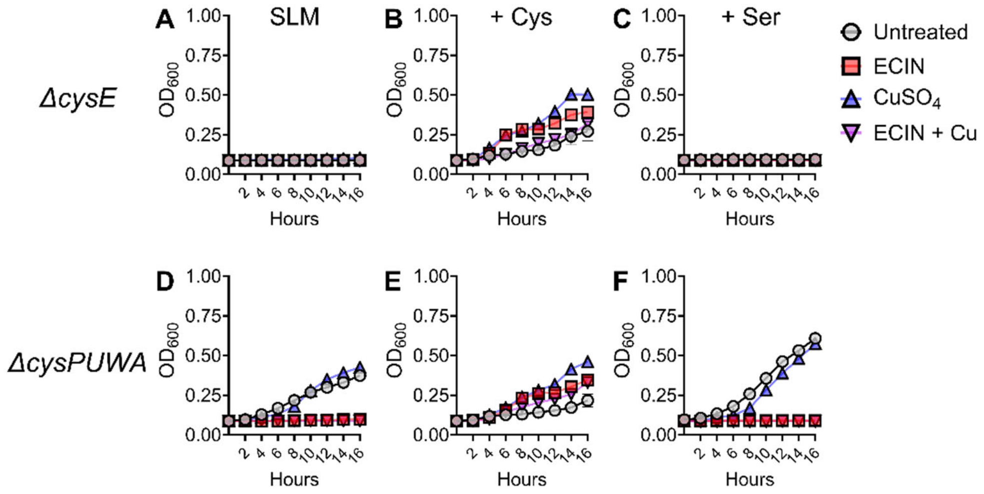

Figure S7. Cysteine rescues the growth of a cysteine auxotroph and a thiosulfate import defective mutant in the presence of ECIN. UPEC strains CFT073Δ*cysE* (**A-C**) and CFT073Δ*cysPUWA* (**D-F**) were cultured in sulfur-limiting medium (SLM) in the presence or absence of ECIN (1.3 µg/mL; 5 µM), copper (5 µM), combination (5 µM each) (**A & D**), L-cysteine (1 mM; **B & E**) or L-serine (1 mM; **C & F**). Optical density was measured over time (n = 9). Error bars represent SEM.

REFERENCES

1. Mobley HL, Green DM, Trifillis AL, Johnson DE, Chippendale GR, Lockatell CV, Jones BD, Warren JW. 1990. Pyelonephritogenic Escherichia coli and killing of cultured human renal proximal tubular epithelial cells: role of hemolysin in some strains. Infect Immun 58:1281-9.
2. Casanova-Hampton K, Carey A, Kassam S, Garner A, Donati GL, Thangamani S, Subashchandrabose S. 2021. A genome-wide screen reveals the involvement of enterobactin-mediated iron acquisition in Escherichia coli survival during copper stress. Metallomics 13.
3. Hyre AN, Kavanagh K, Kock ND, Donati GL, Subashchandrabose S. 2017. Copper Is a Host Effector Mobilized to Urine during Urinary Tract Infection To Impair Bacterial Colonization. Infect Immun 85.
4. Robinson CK, Saenkham-Huntsinger P, Hanson BS, Adams LG, Subashchandrabose S. 2022. Vaginal Inoculation of Uropathogenic Escherichia coli during Estrus Leads to Genital and Renal Colonization. Infect Immun 90:e0053221.
5. Saenkham P, Ritter M, Donati GL, Subashchandrabose S. 2020. Copper primes adaptation of uropathogenic Escherichia coli to superoxide stress by activating superoxide dismutases. PLoS Pathog 16:e1008856.
6. Saenkham-Huntsinger P, Hyre AN, Hanson BS, Donati GL, Adams LG, Ryan C, Londono A, Moustafa AM, Planet PJ, Subashchandrabose S. 2021. Copper Resistance Promotes Fitness of Methicillin-Resistant Staphylococcus aureus during Urinary Tract Infection. mBio 12:e0203821.
7. Saenkham P, Jennings-Gee J, Hanson B, Kock ND, Adams LG, Subashchandrabose S. 2020. Hyperglucosuria induced by dapagliflozin augments bacterial colonization in the murine urinary tract. Diabetes Obes Metab 22:1548-1555.
8. Yep A, McQuade T, Kirchhoff P, Larsen M, Mobley HL. 2014. Inhibitors of TonB function identified by a high-throughput screen for inhibitors of iron acquisition in uropathogenic Escherichia coli CFT073. mBio 5:e01089-13.
9. Subashchandrabose S, Hazen TH, Brumbaugh AR, Himpsl SD, Smith SN, Ernst RD, Rasko DA, Mobley HL. 2014. Host-specific induction of Escherichia coli fitness genes during human urinary tract infection. Proc Natl Acad Sci U S A 111:18327-32.
10. Hyre A, Casanova-Hampton K, Subashchandrabose S. 2021. Copper Homeostatic Mechanisms and Their Role in the Virulence of Escherichia coli and Salmonella enterica. EcoSal Plus 9:eESP00142020.
11. Chen SL, Hung CS, Xu J, Reigstad CS, Magrini V, Sabo A, Blasiar D, Bieri T, Meyer RR, Ozersky P, Armstrong JR, Fulton RS, Latreille JP, Spieth J, Hooton TM, Mardis ER, Hultgren SJ, Gordon JI. 2006. Identification of genes subject to positive selection in uropathogenic strains of Escherichia coli: a comparative genomics approach. Proc Natl Acad Sci U S A 103:5977-82.
12. Andersson P, Engberg I, Lidin-Janson G, Lincoln K, Hull R, Hull S, Svanborg C. 1991. Persistence of Escherichia coli bacteriuria is not determined by bacterial adherence. Infect Immun 59:2915-21.
13. Broberg CA, Wu W, Cavalcoli JD, Miller VL, Bachman MA. 2014. Complete Genome Sequence of Klebsiella pneumoniae Strain ATCC 43816 KPPR1, a Rifampin-Resistant

Mutant Commonly Used in Animal, Genetic, and Molecular Biology Studies. Genome Announc 2.
14. Bakker-Woudenberg IA, van den Berg JC, Vree TB, Baars AM, Michel MF. 1985. Relevance of serum protein binding of cefoxitin and cefazolin to their activities against Klebsiella pneumoniae pneumonia in rats. Antimicrob Agents Chemother 28:654-9.
15. Roosendaal R, Bakker-Woudenberg IA, van den Berg JC, Michel MF. 1985. Therapeutic efficacy of continuous versus intermittent administration of ceftazidime in an experimental Klebsiella pneumoniae pneumonia in rats. J Infect Dis 152:373-8.
16. Roosendaal R, Bakker-Woudenberg IA, van den Berghe-van Raffe M, Michel MF. 1986. Continuous versus intermittent administration of ceftazidime in experimental Klebsiella pneumoniae pneumonia in normal and leukopenic rats. Antimicrob Agents Chemother 30:403-8.
17. Roosendaal R, Bakker-Woudenberg IA, van den Berghe-van Raffe M, Vink-van den Berg JC, Michel MF. 1987. Comparative activities of ciprofloxacin and ceftazidime against Klebsiella pneumoniae in vitro and in experimental pneumonia in leukopenic rats. Antimicrob Agents Chemother 31:1809-15.
18. Pearson MM, Sebaihia M, Churcher C, Quail MA, Seshasayee AS, Luscombe NM, Abdellah Z, Arrosmith C, Atkin B, Chillingworth T, Hauser H, Jagels K, Moule S, Mungall K, Norbertczak H, Rabbinowitsch E, Walker D, Whithead S, Thomson NR, Rather PN, Parkhill J, Mobley HL. 2008. Complete genome sequence of uropathogenic Proteus mirabilis, a master of both adherence and motility. J Bacteriol 190:4027-37.
19. Subashchandrabose S, Smith S, DeOrnellas V, Crepin S, Kole M, Zahdeh C, Mobley HL. 2016. Acinetobacter baumannii Genes Required for Bacterial Survival during Bloodstream Infection. mSphere 1.
20. Piechaud M, Second L. 1951. [Studies of 26 strains of Moraxella Iwoffi]. Ann Inst Pasteur (Paris) 80:97-9.
21. Baumann P, Doudoroff M, Stanier RY. 1968. A study of the Moraxella group. II. Oxidative-negative species (genus Acinetobacter). J Bacteriol 95:1520-41.
22. Fisher MW, Devlin HB, Gnabasik FJ. 1969. New immunotype schema for Pseudomonas aeruginosa based on protective antigens. J Bacteriol 98:835-6.
23. Liu PV, Wang S. 1990. Three new major somatic antigens of Pseudomonas aeruginosa. J Clin Microbiol 28:922-5.
24. Homma JY, Kim KS, Yamada H, Ito M, Shionoya H. 1970. Serological typing of Pseudomonas aeruginosa and its cross-infection. Jpn J Exp Med 40:347-59.
25. Homma JY, Shionoya H, Yamada H, Kawabe Y. 1971. Production of antibody against Pseudomonas aeruginosa and its serological typing. Jpn J Exp Med 41:89-94.
26. Woolfrey BF, Fox JM, Lally RT, Quall CO. 1982. Broth microdilution testing of Pseudomonas aeruginosa and aminoglycosides: need for employing dilutions differing by small arithmetic increments. J Clin Microbiol 16:663-7.
27. Woolfrey BF, Lally RT, Quall CO. 1983. Comparative evaluation of the micro-media system, sceptor, and MIC-2000 microdilution methods for testing Pseudomonas aeruginosa against gentamicin, tobramycin, and amikacin. J Clin Microbiol 17:312-6.
28. Woolfrey BF, Lally RT, Ederer MN, Quall CO. 1984. Evaluation of the automicrobic system for susceptibility testing of Pseudomonas aeruginosa to gentamicin, tobramycin, and amikacin. J Clin Microbiol 19:502-5.

29. Diep BA, Stone GG, Basuino L, Graber CJ, Miller A, des Etages SA, Jones A, Palazzolo-Ballance AM, Perdreau-Remington F, Sensabaugh GF, DeLeo FR, Chambers HF. 2008. The arginine catabolic mobile element and staphylococcal chromosomal cassette mec linkage: convergence of virulence and resistance in the USA300 clone of methicillin-resistant Staphylococcus aureus. J Infect Dis 197:1523-30.
30. Fey PD, Endres JL, Yajjala VK, Widhelm TJ, Boissy RJ, Bose JL, Bayles KW. 2013. A genetic resource for rapid and comprehensive phenotype screening of nonessential Staphylococcus aureus genes. mBio 4:e00537-12.
